# Decomposing decision mechanisms in female substance use disorder: drift diffusion modeling of context-dependent biases in gain and loss processing

**DOI:** 10.1186/s12888-025-07200-9

**Published:** 2025-08-22

**Authors:** Hao Zhang, Gavin Kader, Huoyin Zhang, Haijian Zhao, Wanke Pan, Peng Lei

**Affiliations:** 1 School of Educational Science, Chengdu Normal University, Chengdu, 611130 China; 2 School of Tourism and Cultural Industries, Sichuan Tourism University, Chengdu, 610100 China; 3 Center for Intelligence Economic Science, Southwestern University of Finance and Economics, Chengdu, 611130 China; 4 School of Psychology, Shenzhen University, Shenzhen, 518060 China; 5 School of Psychology, Nanjing Normal University, Nanjing, 210097 China

**Keywords:** Substance use disorder, Intertemporal decision-making, Cognitive mechanisms, Drift diffusion model, Drift rate, Decision threshold

## Abstract

**Background:**

Decision-making impairments are central to substance use disorder (SUD), particularly in evaluating immediate versus delayed outcomes. However, conventional behavioral analyses provide limited insight into underlying cognitive mechanisms. This study applies the Drift Diffusion Model (DDM) to investigate intertemporal decision-making in female SUD across both gain and loss contexts, addressing a significant gap in understanding context-dependent decision processes.

**Methods:**

The study compared 100 females with opioid use disorder to 86 female controls using intertemporal choice tasks in both gain and loss contexts. Participants made choices between smaller-immediate and larger-delayed monetary options across varying magnitudes, delay lengths, and reward differences. Behavioral preferences were analyzed using delay discounting models, while cognitive mechanisms were examined using hierarchical drift diffusion modeling to extract decision parameters (drift rates, thresholds, bias, non-decision time).

**Results:**

Behaviorally, the SUD group showed stronger preferences for immediate rewards in gain scenarios and stronger avoidance of immediate losses in loss scenarios compared to controls. Delay discounting analysis revealed significantly lower discount rates in the SUD group in loss contexts (*p* <.001). DDM analysis demonstrated that the SUD group exhibited lower decision thresholds across contexts, reflecting impulsive decision characteristics. Additionally, they showed lower drift rates in gain scenarios, indicating reduced sensitivity to non-substance rewards, but higher drift rates in loss scenarios, suggesting heightened sensitivity to negative outcomes. These decision patterns varied systematically with monetary and temporal parameters.

**Conclusions:**

This study reveals distinct context-dependent decision biases in female SUD, characterized by computational signatures that differ markedly between gain and loss domains. These findings enhance our understanding of SUD-related decision mechanisms beyond traditional behavioral measures and suggest potential computational targets for individualized assessment and intervention approaches, though these clinical implications remain exploratory and require extensive validation before practical implementation.

## Introduction

### Intertemporal choice and substance use disorder

Intertemporal choice refers to decisions between options with outcomes occurring at different time points, typically immediate and future [20]. This process involves trading immediate gratification for future benefits, influencing health, wealth, and social adaptation while relating to broader societal issues such as financial planning and environmental protection [7, 35]. Consequently, intertemporal choice has emerged as a core topic across psychology, economics, and neuroscience.

Substance use disorder is widely recognized as a disorder of intertemporal choice, with substance use disorder individuals repeatedly choosing immediate substance pleasures despite long-term negative consequences [11]. Behavioral economics research demonstrates that substance use disorder exhibit stronger discounting of future rewards, with future outcomes depreciating more rapidly as delay increases [47]—a behavioral manifestation of high impulsivity and myopic decision preferences [48]. Neuroimaging studies reveal that abnormalities in brain regions such as the prefrontal cortex and striatum may constitute the neural basis for these decision-making deficits [40]. Therefore, investigating intertemporal choice characteristics in substance use disorder individuals and their underlying neurocognitive mechanisms is crucial for understanding substance use disorder development and formulating effective interventions.

The inclusion of female participants in substance use disorder research represents a critical need, as mounting evidence reveals significant sex differences in addiction trajectories, neurobiological mechanisms, and treatment responses. Hormonal factors play a pivotal role, with estrogen fluctuations across the menstrual cycle influencing reward sensitivity and impulsivity [75]. Research by Bobzean et al. [12] demonstrates that estrogen enhances dopamine release in reward circuits, potentially altering temporal discounting patterns in ways distinct from male populations.

Psychosocial factors further differentiate female substance use experiences. Women often initiate substance use in response to trauma, depression, and relationship difficulties, contrasting with male patterns typically driven by sensation-seeking and peer influence [28]. These differential pathways may produce distinct decision-making signatures, with females potentially showing heightened loss sensitivity due to trauma histories and emotional dysregulation.

Empirical evidence supports these theoretical predictions. Studies by Weiss et al. [76] found that women exhibit steeper delay discounting for monetary rewards compared to men, while research by Cross et al. [15] revealed sex-specific patterns in neural activation during intertemporal choice tasks. Critically, most computational modeling studies have been conducted predominantly with male samples [1], leaving female-specific mechanisms largely unexplored and potentially misrepresented by male-derived models.

### Computational approaches to intertemporal decision-making

Traditional models of intertemporal choice, such as the Temporal Discounting Model (TDM), while offering explanatory power for impulsive behavior, have evident limitations. The TDM simplifies decision-making as a static trade-off, failing to explain the dynamic relationship between reward and delay. Although the discount rate (k) indicates how subjective value changes over time, it doesn’t reveal underlying cognitive processes [20]—it can indicate whether an individual is impulsive but cannot explain why or through what mechanisms.

In contrast, cognitive modeling approaches like the Drift Diffusion Model (DDM) offer significant advantages in explaining intertemporal choice behavior. The DDM demonstrates multi-attribute dynamic cognitive processes involved in intertemporal choice, wherein decision-makers evaluate attributes such as time delay and monetary value, forming trade-offs between these attributes [42, 63]. The DDM describes decision-making as evidence accumulation toward choice alternatives [62, 78], following a diffusion process described by the stochastic differential equation Rt + 1 = Rt + v + St, where v is the drift rate and S is mean-zero Gaussian noise.

The DDM has four primary parameters: (1) Drift rate reflects the speed and direction of evidence accumulation, corresponding to relative preference between options; (2) Decision threshold indicates evidence required to make a decision, reflecting caution versus impulsivity; (3) Starting point bias represents initial preference for a particular option; and (4) Non-decision time captures time spent on perceptual encoding and motor execution.

In substance use disorder research, DDM application has revealed important insights into cognitive mechanisms underlying addictive behaviors. Studies on alcohol consumption in rats found inability to delay rewards increased risk of alcohol use disorder [32], while the DDM indicated lower prosociality in individuals with methamphetamine use disorder could be attributed to reduced valuation of others’ benefits [44]. However, other studies found no significant group differences among heroin-substance use disorder mothers and populations with varying alcohol consumption levels [14]. These findings highlight DDM’s value in revealing deficits traditional analyses might miss [77], providing quantitative estimates for understanding decision-making processes in substance use disorder [43, 68].

### Context-dependent decision-making in substance use disorder

Research indicates substance use disorder individuals exhibit unique preference patterns in intertemporal decision-making that vary depending on decision context [10]. While extensive research has examined decisions in gain contexts, less attention has been paid to loss contexts, limiting our understanding of context-dependent substance use disorder-related decision biases.

In gain contexts, substance use disorder individuals typically demonstrate stronger preference for immediate rewards. Kirby and Petry [38] found substance users exhibited steeper delay discounting curves, choosing smaller immediate rewards over larger delayed ones compared to non-substance use disorder individuals—a finding corroborated across various substance use disorder populations [47]. Neuroimaging studies suggest this preference may be associated with reward system hyperactivity and diminished prefrontal control functions [23].

In loss contexts, however, substance use disorder individuals may exhibit distinctive decision preferences. Odum et al. [58] discovered smokers displayed lower discounting rates for losses than gains, suggesting tendencies to avoid delayed losses. This may relate to loss aversion characteristics [36], as substance use disorder individuals are generally more sensitive to negative outcomes and may tend to postpone losses to avoid immediate negative impacts. This context-dependent pattern suggests substance use disorder-related decision biases vary according to whether individuals face potential gains or losses.

Within the DDM framework, several cognitive mechanisms may underlie intertemporal decision biases in substance use disorder individuals across different contexts. In gain contexts, substance use disorder individuals may exhibit lower drift rates, contradicting traditional views of heightened reward sensitivity but aligning with the reward deficiency hypothesis [74]. While substance use disorder’ attention often biases toward substance-related stimuli, their response to natural rewards may be blunted [5, 24], potentially causing slower evidence accumulation when evaluating monetary rewards.

In loss contexts, substance use disorder individuals may exhibit higher drift rates, reflecting heightened sensitivity to negative outcomes related to loss aversion. Their lower discounting rates for delayed losses [10] reflect tendencies to avoid immediate losses, potentially causing more rapid evidence accumulation for loss-related options.

Regarding other DDM parameters, substance use disorder individuals may exhibit lower decision thresholds across contexts, reflecting higher impulsivity [17, 73] related to functional weakening in prefrontal control regions [24, 31]. Their starting point bias may differ from healthy controls, with long-term substance use potentially forming positive expectations for immediate rewards in gain contexts [55] and preferences for delayed losses in loss contexts, possibly related to dopamine dysregulation [8]. Non-decision time likely won’t differ significantly between groups, as chronic substance use typically doesn’t impair basic perceptual-motor abilities [53].

The reward deficiency hypothesis provides a crucial theoretical framework for understanding DDM parameter alterations in substance use disorder. According to Volkow et al. [74], chronic substance use leads to downregulation of dopamine D2 receptors and reduced baseline dopaminergic tone, resulting in diminished sensitivity to natural rewards. Within the DDM framework, this neurobiological alteration should manifest as reduced drift rates for natural reward stimuli in gain contexts, as the blunted reward system requires stronger evidence to drive decisions toward delayed monetary rewards. Conversely, the heightened stress reactivity and negative emotionality characteristic of substance use disorder [41] may enhance sensitivity to negative outcomes, potentially increasing drift rates in loss contexts as individuals become more reactive to potential losses. This dual-system dysregulation—hypoactive reward processing and hyperactive stress response—provides specific predictions for context-dependent DDM parameter patterns in our study.

Intertemporal decisions are also influenced by monetary difference (ΔM) and time difference (ΔT) [25, 65]. Larger monetary differences may increase drift rates toward corresponding options [4], while longer delays may decrease drift rates [16]. These factors may interact, with large monetary differences and small time differences producing highest drift rates (most impulsive decisions), and the opposite combination producing lowest drift rates (most cautious decisions).

Despite the growing body of research on intertemporal decision-making in substance use disorder, several critical gaps remain unaddressed. First, as noted by Vassileva and Conrod [72], the vast majority of studies have focused on male-dominated samples, leaving female-specific mechanisms largely unexplored. Second, Bickel et al. [10] explicitly identified the need for context-dependent analyses, noting that most research has examined only gain contexts while neglecting loss scenarios. Third, recent reviews by Zilverstand et al. [68, 80] have called for computational approaches beyond traditional behavioral measures to understand the underlying cognitive mechanisms of addiction-related decision-making. Finally, MacKillop et al. [47] highlighted the urgent need for research that can inform personalized treatment approaches by identifying specific cognitive targets. The present study directly tries to address these identified gaps by employing computational modeling approach to analyze how the cognitive process and behavior differences comes on female SUD.

### Research hypotheses

Based on the theoretical framework outlined above, this study proposes an integrated set of hypotheses across three domains of inquiry: behavioral preferences, cognitive mechanisms, and task parameters. These hypotheses collectively address the context-dependent nature of decision-making in substance use disorder and the underlying cognitive processes that may drive observed behavioral differences.

Our first set of hypotheses concerns the behavioral preferences exhibited by substance use disorder individuals in intertemporal choice tasks. We predict that in gain contexts, substance use disorder groups will demonstrate a higher preference for immediate rewards compared to normal groups, manifested as a higher tendency to choose immediate rewards and larger delay discounting coefficients (Hypothesis 1a). This prediction aligns with extensive literature documenting impulsive choice patterns in substance use disorder. Conversely, in loss contexts, we hypothesize that substance use disorder groups may exhibit higher levels of delayed loss avoidance, with a higher tendency to choose delayed losses and smaller delay discounting coefficients (Hypothesis 1b). This context-dependent pattern would suggest that substance use disorder-related decision biases manifest differently depending on whether individuals face potential gains or losses.

The second set of hypotheses addresses the cognitive mechanisms underlying these behavioral preferences, as captured by DDM parameters. Regarding evidence accumulation processes, we hypothesize that in gain contexts, substance use disorder groups will exhibit lower drift rates compared to control groups, reflecting reduced sensitivity to non-substance rewards consistent with the reward deficiency hypothesis (Hypothesis 2a). In contrast, within loss contexts, substance use disorder groups will demonstrate higher drift rates, reflecting heightened sensitivity to negative outcomes. Furthermore, we propose that in both gain and loss contexts, substance use disorder groups will have lower decision thresholds, reflecting the impulsive characteristics of their decision-making process and reduced evidence requirements before committing to a choice (Hypothesis 2b). Regarding initial biases, we predict that compared to normal groups, substance use disorder individuals’ starting points will be more biased toward immediate options in gain contexts and toward delayed options in loss contexts, reflecting pre-existing tendencies shaped by reinforcement learning histories (Hypothesis 2c). Finally, we hypothesize that there will be no significant difference in non-decision time between normal and substance use disorder groups, as basic perceptual and motor processes are typically unaffected by substance use disorder (Hypothesis 2d).

Our third set of hypotheses concerns how key task parameters influence the decision process. We hypothesize that the larger the monetary difference (ΔM), the higher the participant’s drift rate, reflecting a faster convergence toward the corresponding option when faced with larger monetary temptations (Hypothesis 3a). This effect is expected to be present in both groups but potentially more pronounced in substance use disorder individuals. Regarding temporal factors, we predict that the larger the time difference (ΔT), the lower the participant’s drift rate, reflecting increased caution in the decision process when faced with longer delay times (Hypothesis 3b). Furthermore, we hypothesize that there exists an interaction between monetary difference and time difference (Hypothesis 3c). Specifically, when the monetary difference is large and the time difference is small (representing large short-term temptations), participants’ drift rates will be highest, reflecting the most impulsive decision process. Conversely, when the monetary difference is small and the time difference is large (representing small long-term returns), participants’ drift rates will be lowest, reflecting the most cautious decision process. This interaction may be particularly informative for understanding the computational mechanisms underlying temporal discounting in substance use disorder.

These hypotheses collectively provide a comprehensive framework for investigating the context-dependent nature of decision-making deficits in substance use disorder and their underlying cognitive mechanisms. By testing these predictions, this study aims to advance our understanding of substance use disorder-related decision biases beyond traditional behavioral measures and reveal the computational processes that drive maladaptive choice patterns.

This study advances substance use disorder research through four key innovations: (1) focusing on female opioid substance use disorder, an understudied population typically overshadowed by male-dominated research; (2) examining intertemporal decision-making in both gain and loss contexts, revealing context-dependent decision patterns critical for comprehensive intervention development; (3) employing the Drift Diffusion Model to analyze cognitive mechanisms underlying substance use disorder-related decision biases, providing finer-grained analysis than traditional behavioral approaches; and (4) investigating how monetary and time differences influence decision processes, offering new perspectives on value computation and time perception in substance use disorder. These contributions may inform development of targeted assessment tools and intervention strategies for substance use disorder treatment.

## Methodology

### Subjects

This study employed a convenience sampling method to recruit participants in two stages. The first stage involved an intertemporal decision-making experiment with non-substance use disorder participants to provide a reference for comparing behavioral characteristics with the substance use disorder group. The control group consisted of 90 female university students recruited from a university in Chengdu in November 2022. Inclusion criteria were: (1) age 18–25 years; (2) no history of mental illness; and (3) no history of substance abuse. Exclusion criteria included: (1) severe physical illness; and (2) recent use of medications that could affect cognitive function.

The second stage involved an intertemporal decision-making experiment with a female substance use disorder group, conducted in July 2023 at a women’s substance rehabilitation center in Sichuan Province. A total of 100 female substance use disorder participants were recruited. Inclusion criteria were: (1) meeting the DSM-5 diagnostic criteria for substance use disorder; (2) age 18–32 years; and (3) at least primary school education (not illiterate). Exclusion criteria included: (1) history of severe mental illness (e.g., schizophrenia, bipolar disorder); (2) severe physical illness; and (3) obvious cognitive impairment.

The age difference between groups (control: 18–25 years; SUD: 18–32 years) reflects practical constraints of recruiting from different populations. University students typically fall within a narrower age range, while treatment center patients represent a broader age spectrum. To address potential age-related confounding effects on temporal discounting [26], we conducted supplementary analyses controlling for age as a covariate. Results remained consistent when age was included as a covariate in our models, suggesting that our findings are not primarily driven by age differences.

The sample size was determined based on effect size estimates from similar previous studies and power analysis (G*Power software, α = 0.05, 1-β = 0.80, expected effect size d = 0.3), which indicated a minimum requirement of 82 participants per group. Both experimental groups in this study met the minimum sample size requirement.

The incentive method for participants involved randomly selecting a number corresponding to a decision task after completing the experiment, with the reward form determined by the choice made in the corresponding task. For the control group, if participants chose immediate rewards in the selected intertemporal decision task, compensation was settled on the same day; if future rewards were chosen, compensation was provided after the corresponding delay period. The average earnings for university student participants were 45.75 (± 14.25) RMB. For the substance use disorder group, due to management requirements of the treatment center, experimental incentives were replaced with equivalent daily necessities (such as tissues, socks, gloves, etc.), distributed either on the same day or within one week. The average earnings for the substance use disorder group were equivalent to 25.25 (± 14.75) RMB in goods. The difference in incentive types between groups was mandated by institutional policies at the treatment facility, which prohibits cash distribution to residents. While this introduces a potential confounding factor, several considerations suggest minimal impact on decision validity: (1) participants were informed of the incentive structure before beginning the experiment, ensuring informed decision-making; (2) the equivalent monetary value of goods was maintained; (3) previous research indicates that hypothetical and real monetary rewards produce similar temporal discounting patterns [33]; and (4) the relative nature of choices (immediate vs. delayed) remained constant across groups, preserving the fundamental decision structure under investigation.

All participants were informed of the incentive method before the experiment began, but not of the specific calculation method, to enhance the authenticity of decision-making during participation. For the substance use disorder group, in addition to the intertemporal decision-making experiment, substance use disorder-related demographic variables were collected, including age, education level, and type of substance used.

Several demographic differences between groups warrant consideration. The SUD group was older (M = 24.8 vs. 19.3 years) and had lower educational attainment, factors that could independently influence temporal discounting [18, 26]. Additionally, the incentive structure differed between groups (cash vs. goods), potentially altering motivational salience. While these differences reflect the practical realities of studying clinical populations, they represent potential confounding variables that could contribute to observed group differences beyond substance use disorder per se.

This study strictly adhered to the ethical requirements for human subject research as outlined in the Declaration of Helsinki and was approved by the Research Ethics Subcommittee of the Sichuan Psychological Society (No. 2022102). All participants signed informed consent forms and were informed that they could withdraw from the experiment at any stage and had the right to request or discard their experimental data.

### Experiment design

To better explore the research hypotheses, this study aimed to investigate individual behavioral preference differences when facing gains and losses across various time dimensions through a series of intertemporal decision tasks in both gain and loss contexts. Previous research has found that the general population typically prefers larger future gains and smaller immediate losses, consistent with the rational preference for maximizing gains and minimizing losses (Kirby & Herrnstein, 1995; Tom et al., [71]. However, due to the impact of substances on reward circuits, it is theoretically hypothesized that substance use disorder groups might exhibit stronger preferences for smaller immediate gains and larger future losses [9]. These preferences can be reflected not only in decision outcomes but also captured through decision processes (e.g., reaction times) [57]. Other studies have shown that intertemporal decision preferences vary with different reward magnitudes, baseline delays, and delay lengths [27]. Substance use disorder groups may exhibit significant differences in behavioral tendencies from the general population in these contexts [79]. Therefore, this study adopted a series of task designs to effectively uncover unique behavioral indicators of substance use disorder groups across multiple aspects of intertemporal decision-making.

The specific experimental design encompassed intertemporal decision tasks in both gain and loss contexts, systematically examining multiple decision context variables including time baseline, delay length, reward magnitude, and delayed reward difference rate. Regarding time baseline differences, research has shown that current and delayed baselines influence individual intertemporal preferences. Differences in delay length also impact decision-making, with short-term and long-term delays typically eliciting different decision patterns [45]. Reward magnitude is another important moderating factor, with individuals often exhibiting lower delay discounting rates when facing larger rewards [27, 64]. Finally, the difference rate of delayed rewards determines the attractiveness of choosing smaller, earlier rewards.

This study’s design considered the aforementioned contextual variables, specifically including: two-time baselines (current and 100 days later), two delay lengths (7 days and 30 days), three reward magnitudes (100/400/1000 tokens), and six delayed reward difference rates (5%/10%/15%/20%/25%/30%). The full combination of these variables ultimately formed 144 distinct decision tasks. By systematically manipulating and examining these variables, it is expected to comprehensively capture behavioral differences and unique cognitive patterns in intertemporal decision-making between the general population and substance use disorder groups.

**Fig. 1:**
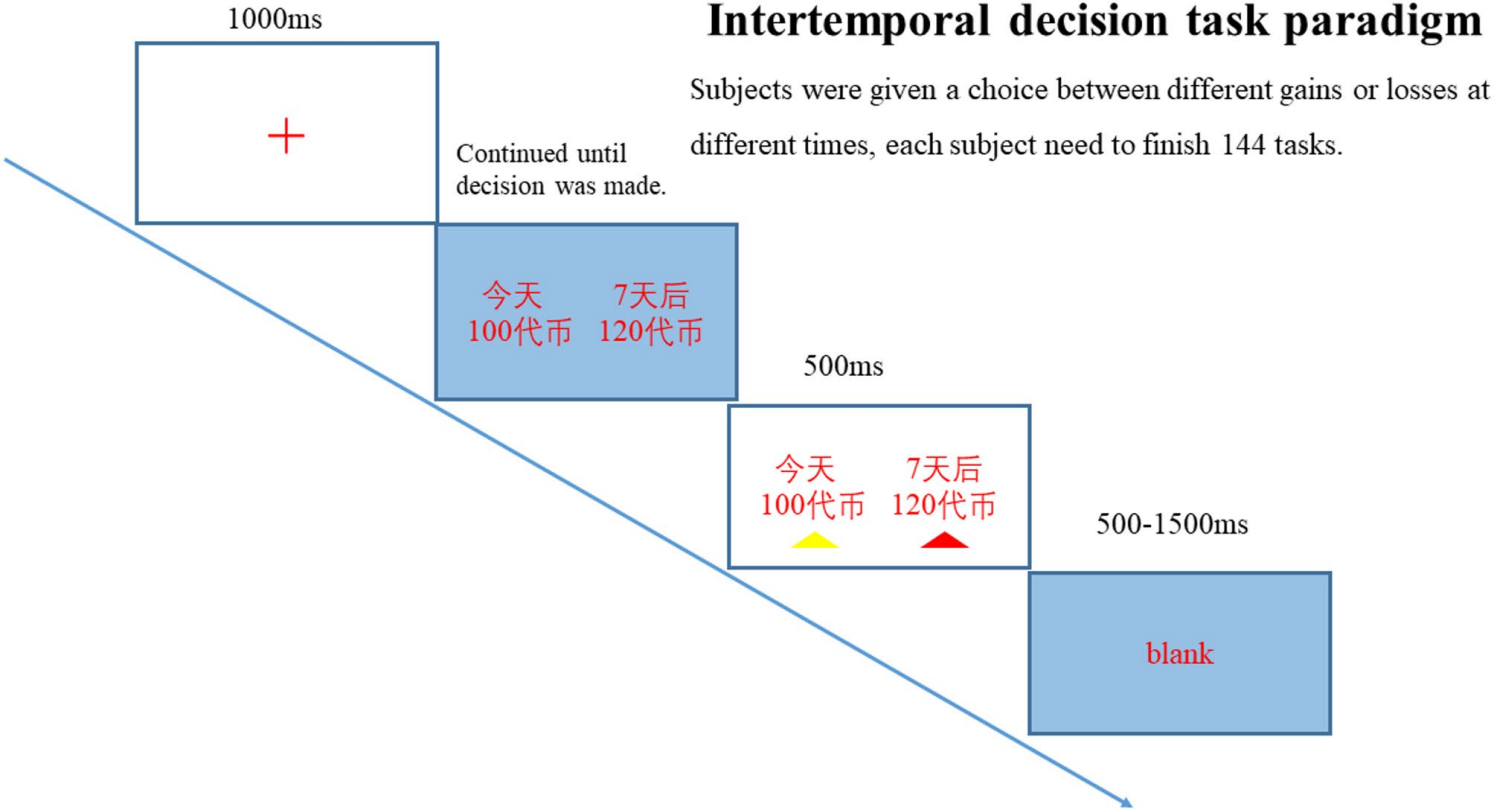
Flow chart of intertemporal decision experiment

The experimental procedure is illustrated in Fig. 1. All 144 decision tasks were presented in fully randomized order for each participant to control for potential order effects, learning, and fatigue. The randomization included both task content (monetary amounts, delays) and spatial positioning (left/right option placement). To further minimize fatigue effects, participants were offered a brief rest period every 36 trials (approximately every 10 min). The extensive randomization ensures that any systematic learning or adaptation effects would be distributed equally across experimental conditions and groups. In each decision task, participants were first presented with a red cross screen for 1000ms to focus their attention. Subsequently, current gain (loss) and delayed gain (loss) options were randomly presented on the left and right sides of the screen. Both the content and position of the options were randomized to ensure that each decision task was conducted independently as much as possible. Participants pressed the “F” key to select the left option and the “J” key to select the right option, with a maximum decision time of 10000ms. The task progressed to the next screen once the participant made a choice within the specified time or when the decision time exceeded 10000ms. If a participant failed to make a decision within 10000ms, that record was excluded. The next screen displayed a small red triangle indicating the participant’s choice, lasting for 500ms. This was followed by a random blank screen lasting 500ms-1500ms, completing one decision task before randomly proceeding to the next.

The experimental program was coded using E-Prime 3.0 and conducted on identical laptop computers. Participants entered standardized behavioral laboratories in batches, with no communication or interaction between them, and no mutual interference. After completing the experiment, participants drew random numbers in an adjacent rest area to determine their reward earnings.

### Drift diffusion model

This study employed HDDM (Hierarchical Drift Diffusion Model), a Python-based tool [78], for data modeling and analysis. HDDM is a Bayesian-based drift diffusion model that allows researchers to estimate latent parameters in cognitive decision processes, such as drift rate, decision threshold, and non-decision time. Through this method, we can meticulously investigate how substance use disorder states affect individual decision-making processes and reveal potential mechanisms by which substance use disorder influences cognitive functions under different decision frameworks. Furthermore, this study will examine the relationship between substance use disorder severity and decision parameters, aiming to provide empirical evidence for further understanding addictive behaviors and their impact on cognitive functions.

According to Gui et al. [29, 67], we primarily focus on the effects of monetary differences and time differences on information accumulation speed in intertemporal decision-making. The mathematical form is as follows:

$$\begin{aligned} v&={\beta\:}_{0}+{\beta}_{reward}*MoneyDiff+{\beta}_{time}\\& \quad * DelayDiff +{\beta}_{time-base}*DelayBaseline\#\end{aligned}$$

Where the drift rate (v) is the core parameter in the drift diffusion model, reflecting the speed of information accumulation during the decision process, with the upper boundary representing delayed response. In this study, the drift rate is modeled as a composite effect of several factors, including baseline drift rate (β₀), the impact of monetary difference on drift rate (β_reward_ * MoneyDiff), and the impact of time difference on drift rate (β_time_ * DelayDiff). Monetary difference (MoneyDiff) represents the monetary disparity between two options in intertemporal decision-making. In the model, the coefficient of monetary difference (β_reward_) quantifies the weighted impact of monetary difference on information accumulation speed. Time difference (DelayDiff) reflects the temporal disparity between two options in intertemporal decision-making. The coefficient of time difference (β_time_) reveals the weighted impact of time difference on information accumulation speed. Additionally, the term β_(time−base)_ * DelayBaseline represents the influence of a baseline temporal parameter on the drift rate. DelayBaseline serves as a fixed reference point that affects decision-making processes. This component captures the inherent cognitive delay that, while not directly tied to immediate choices, impacts the evaluation of future rewards. The coefficient β_(time−base)_ reveals how this baseline reference modulates the drift rate, contributing to our understanding of the cognitive mechanisms involved in intertemporal choices.

This study adopts a hierarchical model to analyze cognitive differences among participants with varying degrees of substance use disorder under different decision frameworks. Each participant has their own drift rate coefficients in two decision scenarios, including baseline drift rate, monetary difference impact coefficient, and time difference impact coefficient. For parameter priors, this study uses HDDM’s default prior settings, which are weakly informative priors (for details, refer to Wiecki et al., [78]. Additionally, HDDM employs the MCMC method to fit the model. To ensure adequate estimation of posterior parameter distributions, this study set 10,000 samples with a burn-in period of 5,000 and used 4 chains. All parameters’ Rhat values were less than or equal to 1.01, indicating model convergence [22].

### Statistical analysis

The main statistical analyses in this study include:1) Independent samples t-tests for comparing intertemporal decision-making behavioral outcomes between groups. 2)Independent samples t-tests for comparing discounting factor coefficients between groups. 3)Multivariate analysis of variance (MANOVA) for comparing Drift Diffusion Model (DDM) parameters between groups, examining both main effects and interaction effects. To address the issue of multiple comparisons, Bonferroni correction was applied to adjust p-values. Effect sizes are reported using Cohen’s d for t-tests and partial η² for MANOVA. All statistical analyses were conducted using SPSS.

## Result

### Descriptive statistic

Four university students were excluded due to equipment issues, resulting in a final sample of 100 female substance use disorder (mean age 24.83 ± 5.5 years) and 86 female university students (mean age 19.3 ± 0.95 years). In the substance use disorder group, 2% had bachelor’s degrees, 26% had associate degrees, 50% had high school diplomas, and 22% had middle school education or below. The primary substances used were methamphetamine and heroin. The control group primarily comprised students majoring in education, psychology, and biology, most are freshman in campus. All participants were in good physical health, had normal color vision, and were right-handed.

**Fig. 2:**
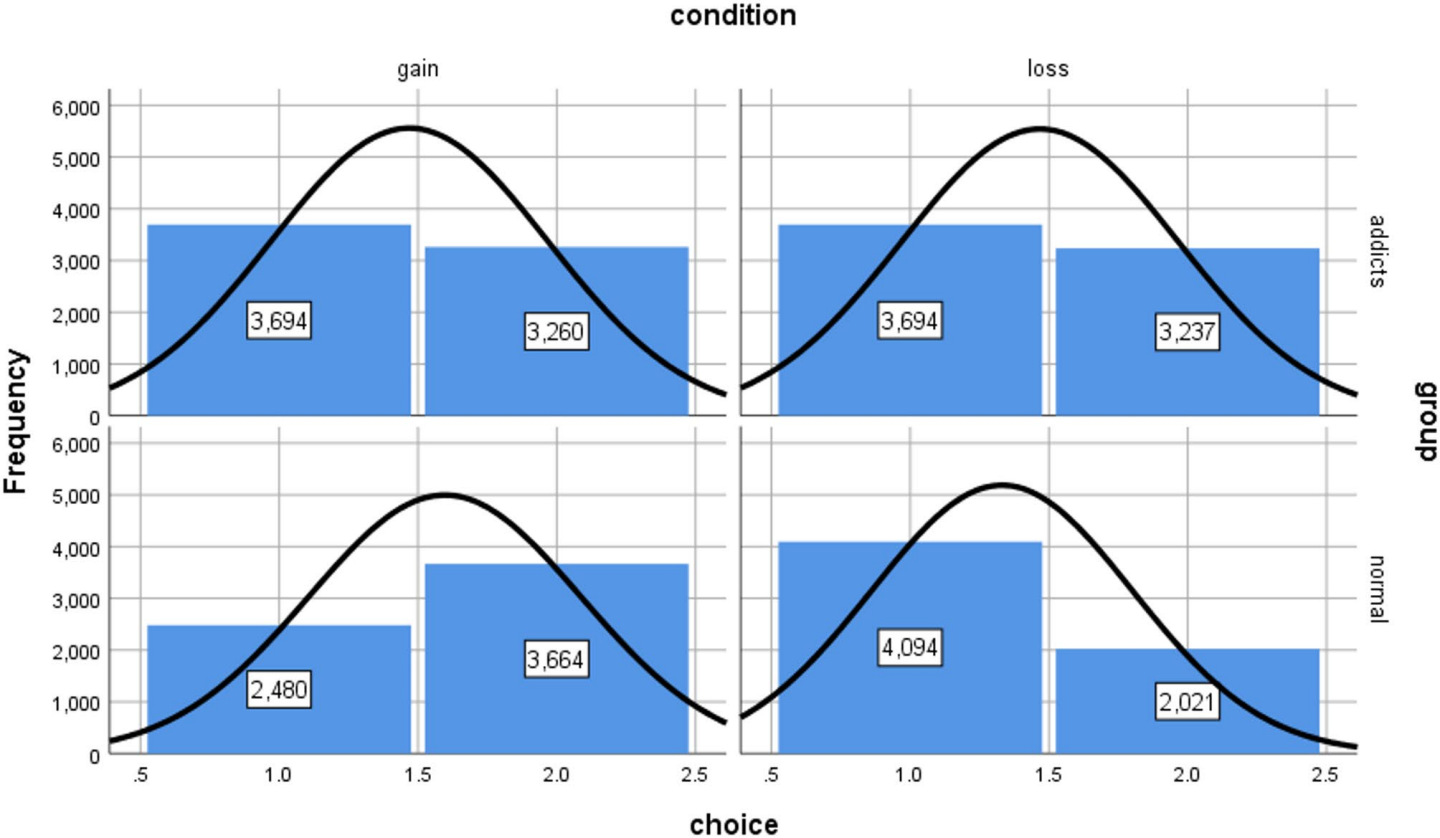
Distribution characteristics of decision results between the addict group and the normal group under different intertemporal decision situations

**Fig. 3:**
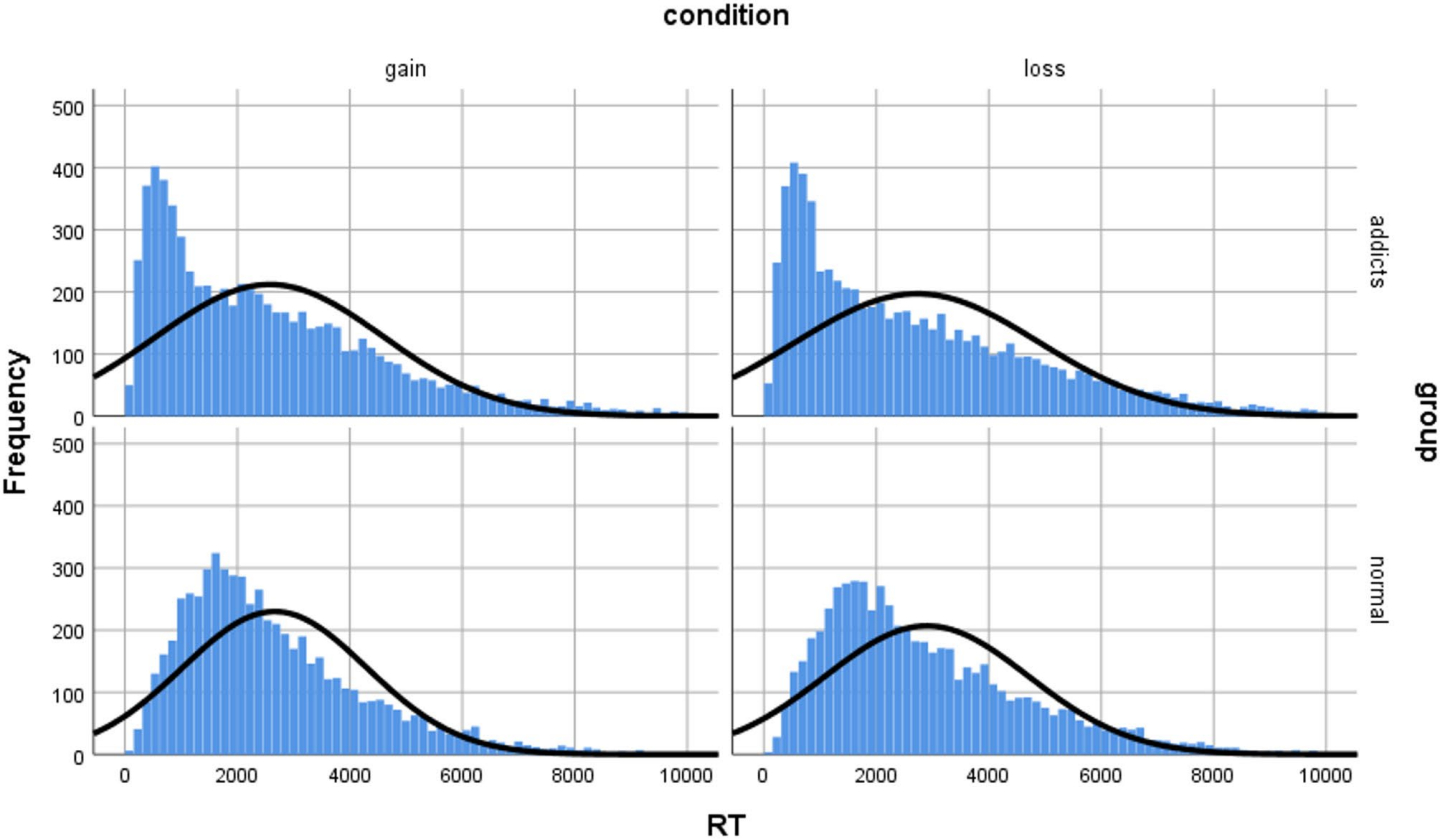
Distribution characteristics of response time between the addict group and the normal group under different intertemporal decision situations

To ensure data quality, we excluded response times below 100ms, removing 640 observations (2.39% of total). The final dataset included 13,885 decision records from the substance use disorder group and 12,559 from the control group. Figures 2 and 3 illustrate the distribution characteristics of decision outcomes and response times across groups and contexts. The control group predominantly chose delayed options in gain scenarios and immediate options in loss scenarios, reflecting rational maximizing/minimizing strategies. In contrast, the substance use disorder group more frequently chose immediate options in both contexts, indicating stronger impulsivity and difficulty inhibiting immediate choices. Response time distributions showed the control group approximated normal distributions in both contexts, while the substance use disorder group showed left-skewed distributions with predominantly shorter decision times, further reflecting impulsive decision-making tendencies.

### Delay discount rate analysis

We calculated individual discount rates using Mazur’s [49] hyperbolic discounting function:

$$ V =A/(1+kD)$$

where V represents the subjective value of the smaller immediate reward, A is the larger delayed reward value, D is the delay length, and k is the discount rate. Higher k values indicate stronger preference for immediate rewards.

**Fig. 4:**
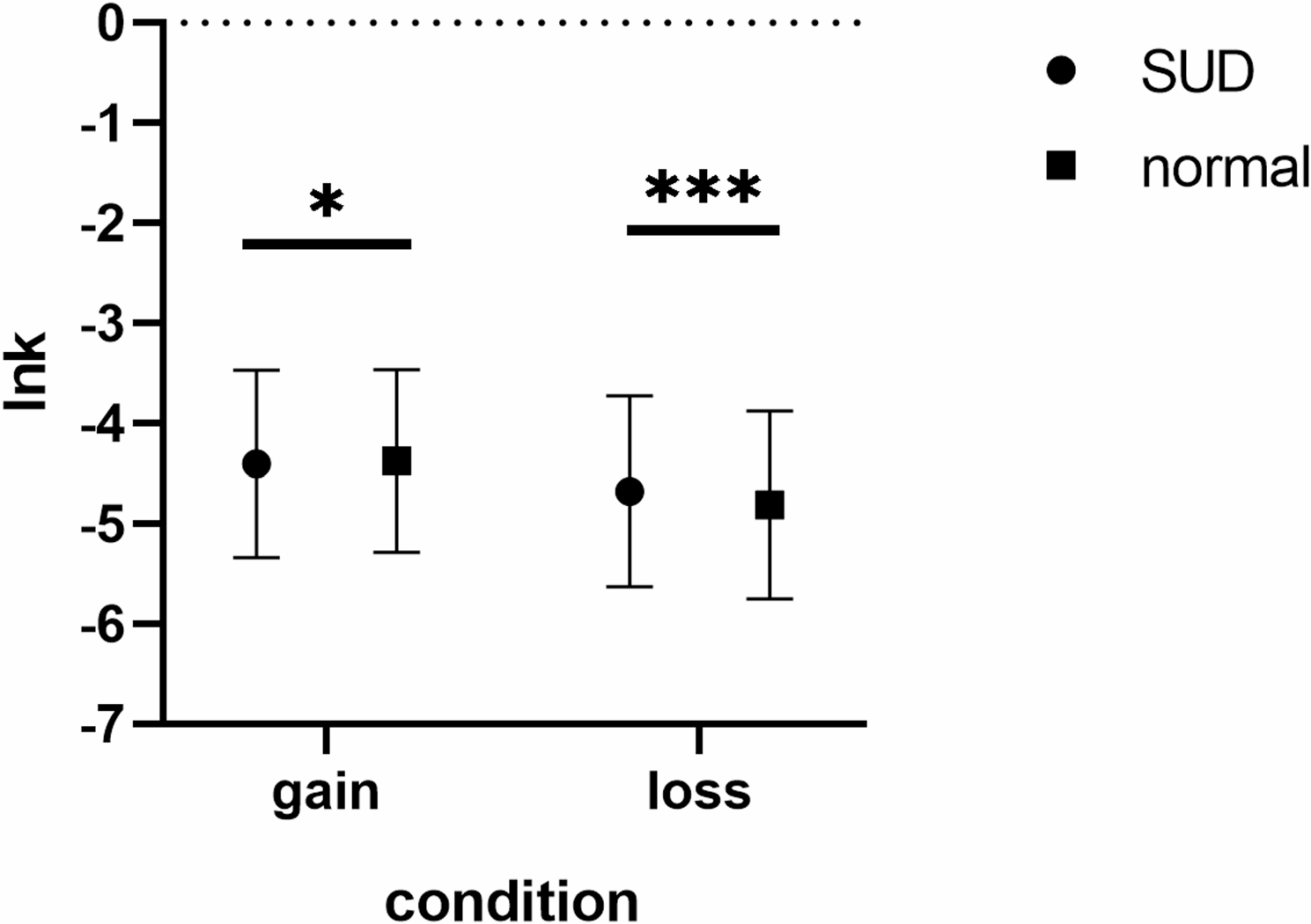
Comparison of mean logarithmic delay discount coefficients of different groups in intertemporal gain (loss) decision making

Discount coefficients were estimated using nonlinear least squares in Stata and natural log-transformed (lnk) to approximate a normal distribution. Independent sample t-tests compared lnk values between groups in both gain and loss contexts (Fig. 4).

In gain scenarios, the substance use disorder group showed a numerically higher mean lnk value (M = 4.40, SD = 0.33) than the control group (M = 4.37, SD = 0.28), but this difference did not reach statistical significance, t(6922) = -1.35, *p* =.089. Therefore, Hypothesis 1a predicting significantly higher delay discounting in gain contexts was not supported by our data, though the directional trend was consistent with theoretical predictions.

In loss scenarios, the substance use disorder group showed a significantly lower mean lnk value (M = 4.67, SD = 0.41) than the control group (M = 4.81, SD = 0.37), t(5256) = 5.13, *p* <.001. This significant difference indicates substance use disorder exhibit higher levels of delayed loss avoidance, potentially reflecting dysfunction in risk avoidance mechanisms or an adaptive strategy to prevent further losses.

### DDM parameters analysis

To systematically evaluate our theoretical predictions, we examine DDM parameters in relation to our pre-registered hypotheses. Our analysis addresses Hypotheses 2a-2d regarding cognitive mechanisms, and Hypotheses 3a-3c regarding task parameter effects.

To explore the similarities and differences in cognitive processes between different participant groups under two decision frameworks, this study ensured effective control over the effects of monetary value differences and time differences on information accumulation speed in intertemporal decision-making. We employed a model comparison approach, examining three models: Model 1 considered only the effect of value differences on information accumulation; Model 2 considered only the effect of time differences; and Model 3 considered the joint effect of both value and time differences.

The model comparison results (Table 1) showed that value differences and time delays had similar effects on drift rates, resulting in similar DIC values for Models 1 and 2 (DIC difference < 10). However, Model 3, which simultaneously considered the joint effect of value differences and time delays on drift rates, showed a significantly lower DIC value, indicating that both factors significantly influence drift rates.

**Table 1:** Model comparison result

	$\:{v}_{{\beta\:}_{rward}}$	$\:{v}_{{\beta\:}_{time}}$	$\:{v}_{{\beta\:}_{base-time}}$	$\:DIC$	$\:{\Delta\:}DIC$
Model 1	√			46,403	
Model 2		√		46,399	4
Model 3	√	√		45,852	547
Model 4	√	√	√	45,660	252

Note: there is no improvement for the model with interaction between $\:{v}_{{\beta\:}_{time}}$ and $\:{v}_{{\beta\:}_{base-time}}$.$\:{\Delta\:}DIC$=2, given that Model 5 (with interaction) has DIC 45,598

**Fig. 5:**
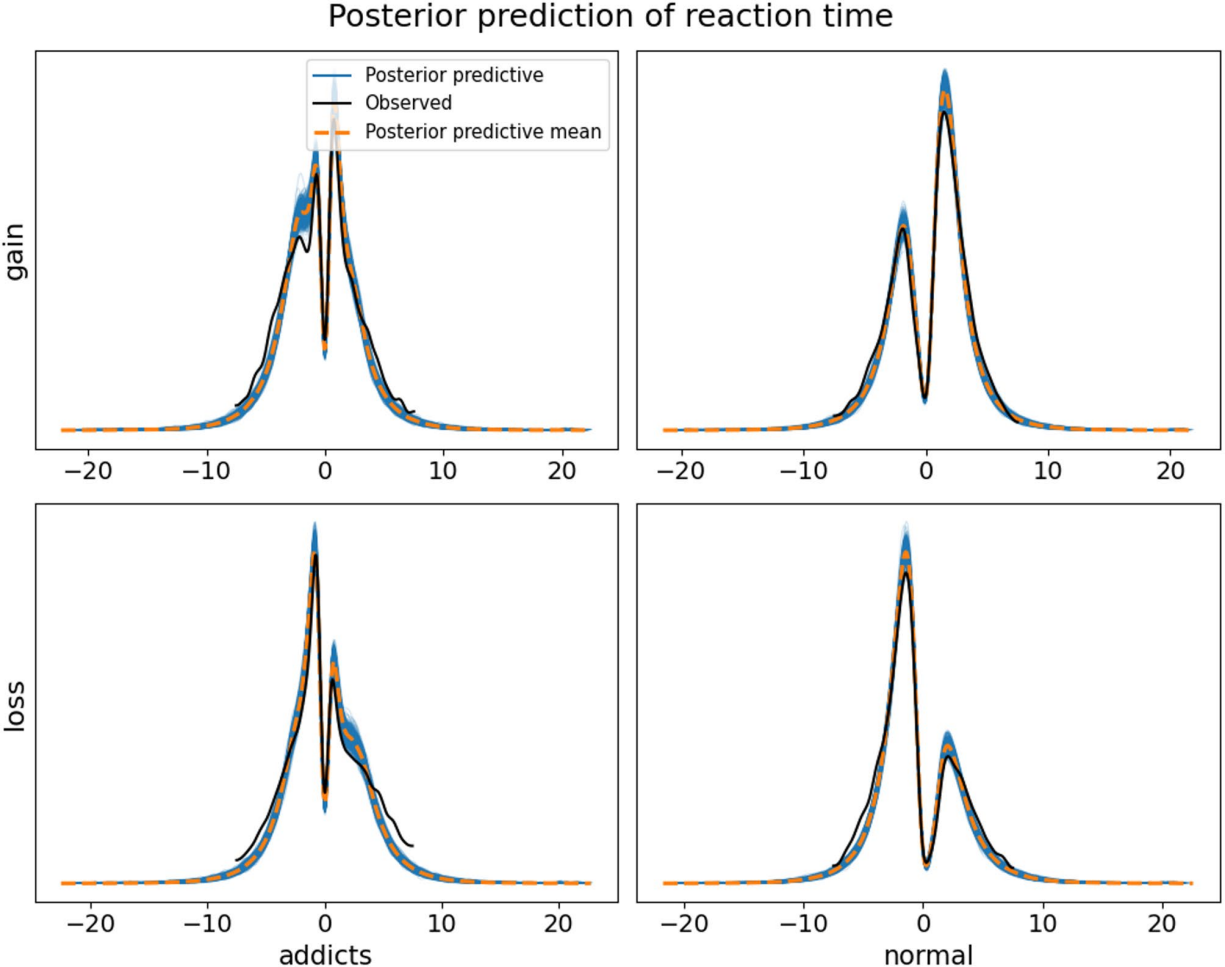
Posterior prediction test

While model comparison provided a relative assessment of different models’ fitting performance, it did not directly ensure that the models accurately captured the response characteristics of different participant groups under various experimental conditions. To provide an absolute evaluation of the models’ predictive ability, we employed posterior predictive methods. We simulated reaction time and behavioral data for different participant groups under various experimental conditions and compared these with actual observed data. The results of the posterior predictive (Fig. 5) analysis indicated that the model effectively predicted the distribution characteristics of reaction times under both loss and gain conditions for healthy and substance use disorder groups.

**Fig. 6:**
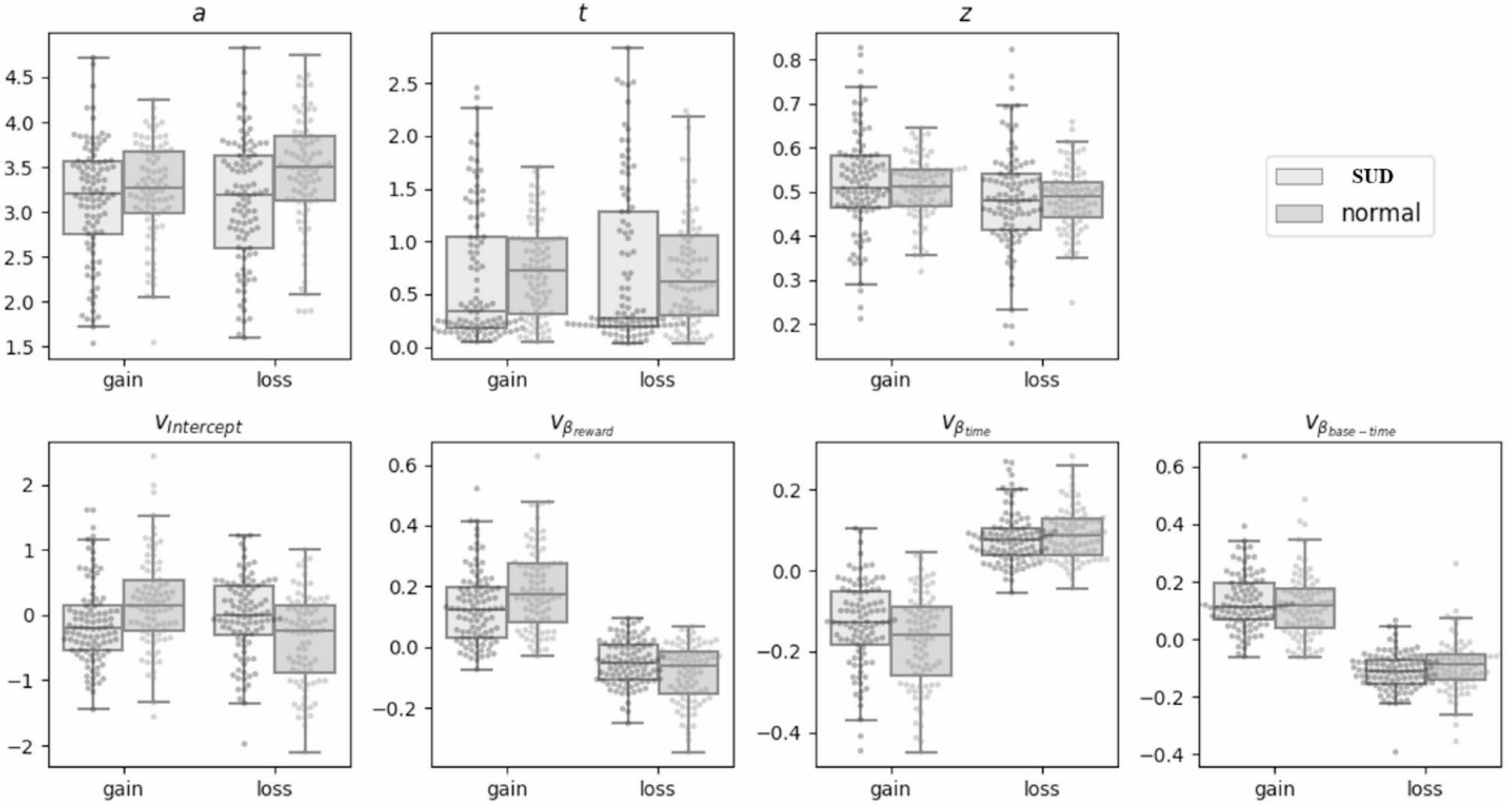
Parameter analysis results of drift diffusion model

To further examine differences in individual parameters between participant groups (between-group) and experimental contexts (within-group), we conducted an in-depth analysis using multivariate analysis of variance (MANOVA). The main parameter differences are shown in Fig. 6.

#### Decision boundary (a)

Analysis revealed a significant main effect of decision context (F(1, 181) = 13.891, *p* <.001), with participants establishing more conservative thresholds in loss scenarios (M = 3.275, 95% CI [3.179, 3.371]) compared to gain scenarios (M = 3.168, 95% CI [3.079, 3.257], d = 0.277). Furthermore, a significant group × context interaction emerged (F(1, 181) = 16.632, *p* <.001), wherein control participants exhibited substantially higher decision boundaries in loss scenarios (M = 3.450, 95% CI [3.310, 3.589]) relative to substance use disorder participants (M = 3.100, 95% CI [2.968, 3.231], t(181) = 3.600, *p* <.001, d = 0.909). Notably, this group difference was absent in gain scenarios (*p* =.199), suggesting that while all participants demonstrated increased caution when facing potential losses, this adaptive response was significantly attenuated in the substance use disorder population.

#### Decision Bias (z)

The decision context significantly influenced starting point bias (F(1, 181) = 7.006, *p* =.009), with participants exhibiting an a priori preference toward delayed responses in gain conditions (M = 0.512, 95% CI [0.498, 0.526]) and toward immediate responses in loss conditions (M = 0.484, 95% CI [0.470, 0.499], d = 0.197). Neither the group main effect nor the interaction reached statistical significance (both *p* =.737), indicating that this context-dependent bias manifested similarly across both populations.

#### Non-Decision time (t)

Neither the main effects of group and context nor their interaction reached statistical significance (all *p* >.05), suggesting that basic perceptual-motor processes remained intact and comparable across conditions.

#### Baseline drift rate (β₀)

A pronounced context effect emerged (F(1, 181) = 17.578, *p* <.001), characterized by evidence accumulation toward delayed options in gain scenarios (M = 0.101, 95% CI [0.006, 0.196]) and toward immediate options in loss scenarios (M = -0.245, 95% CI [-0.339, -0.152]). This main effect was qualified by a significant group × context interaction (F(1, 181) = 15.116, *p* <.001). Subsequent analyses revealed that while control participants demonstrated robust sensitivity to contextual shifts (F(1, 181) = 30.797, *p* <.001), substance use disorder participants exhibited markedly diminished contextual differentiation (F(1, 181) = 0.049, *p* =.824). This pattern suggests fundamental differences in context-dependent information processing between groups, with substance use disorder individuals showing impaired adaptation to changing decision frameworks.

#### Monetary difference effect (β_reward)

Monetary differences exerted significantly different influences across contexts (F(1, 181) = 304.614, *p* <.001), with larger rewards facilitating evidence accumulation toward delayed options in gain scenarios (M = 0.161, 95% CI [0.142, 0.179]) while promoting drift toward immediate options in loss scenarios (M = -0.068, 95% CI [-0.081, -0.056]). A significant interaction (F(1, 181) = 13.441, *p* <.001) revealed differential processing between groups. In gain scenarios, control participants demonstrated heightened sensitivity to monetary differences (M = 0.191, 95% CI [0.164, 0.218]) compared to substance use disorder participants (M = 0.130, 95% CI [0.105, 0.156], t(181) = 3.215, *p* =.002, d = 0.343). Conversely, in loss scenarios, control participants exhibited stronger responses to monetary variations (M = -0.086, 95% CI [-0.104, -0.069]) than substance use disorder participants (M = -0.051, 95% CI [-0.067, -0.034], t(181) = -2.898, *p* =.004, d = -0.201). These findings collectively indicate attenuated sensitivity to monetary differences among substance use disorder individuals, potentially reflecting reward processing alterations.

#### Time difference effect (β_time)

Analyses yielded significant effects for group (F(1, 181) = 4.083, *p* =.045), context (F(1, 181) = 458.395, *p* <.001), and their interaction (F(1, 181) = 4.841, *p* =.029). Across conditions, control participants demonstrated stronger responses to time differences (M = -0.041, 95% CI [-0.052, -0.030]) compared to substance use disorder participants (M = -0.026, 95% CI [-0.036, -0.016], t(181) = -2.021, *p* =.045, d = -0.103). The robust context effect revealed that time differences prompted evidence accumulation toward delayed options in loss scenarios (M = 0.083, 95% CI [0.074, 0.093]) but toward immediate options in gain scenarios (M = -0.150, 95% CI [-0.166, -0.135], t(181) = 21.410, *p* <.001, d = 1.590). The interaction further clarified that in gain scenarios, control participants exhibited enhanced sensitivity to temporal factors (M = -0.170, 95% CI [-0.193, -0.147]) relative to substance use disorder participants (M = -0.131, 95% CI [-0.153, -0.109], t(181) = -2.421, *p* =.016, d = -0.267), whereas this group difference disappeared in loss scenarios (*p* =.352). This pattern suggests that while substance use disorder individuals demonstrate generally attenuated temporal sensitivity, this deficit normalizes when processing potential losses.

## Discussion

### Contextual decision-making patterns in substance use disorder

Our investigation into intertemporal choice processes reveals that substance use disorder individuals demonstrate distinct, context-dependent decision-making patterns that vary significantly between gain and loss domains. In gain scenarios, the substance use disorder group exhibited stronger preferences for immediate rewards compared to controls, as evidenced by their higher selection frequency of immediate options across time baselines and elevated discount coefficients. Conversely, in loss scenarios, substance use disorder individuals displayed heightened delayed loss avoidance, selecting delayed losses more frequently than controls—a pattern further corroborated by discount model analysis. These findings support our context-dependency hypotheses (1a and 1b), demonstrating that substance use disorder-related decision biases manifest differently depending on outcome valence.

These results both confirm and extend previous research. The preference for immediate gratification in gain contexts aligns with established literature documenting heightened delay discounting in substance use disorder populations [11, 47]. This pattern reflects the cognitive dissonance and self-regulation deficits characteristic of substance use disorder [6, 73]. Neurobiologically, excessive activation of reward circuits (ventral striatum, orbitofrontal cortex) coupled with diminished prefrontal regulatory function compromises the ability to inhibit immediate reward-seeking behavior, resulting in myopic decision patterns [24]. This dual-system dysregulation—heightened reward reactivity and impaired cognitive control—appears central to substance use disorder-related impulsivity [6].

However, our findings regarding loss contexts represent a novel contribution to the literature. The pronounced delayed loss avoidance exhibited by substance use disorder individuals suggests that decision-making biases in substance use disorder are fundamentally context-dependent, with distinctive manifestations across different outcome valences. This aligns with theoretical frameworks positing that multiple factors—including outcome valence, emotional states, and environmental cues—modulate decision processes in substance use disorder [24]; Loewenstein [46],. Goldstein and Volkow’s (2011) model proposes that substance use disorder-related decision deficits stem from dysregulation across multiple neural circuits (reward, habit, motivation, and control networks), with circuit activity differentially modulated by contextual factors. Similarly, Loewenstein’s (1996) “visceral influences” theory contends that decision preferences are shaped by internal states (hunger, fatigue, anxiety), which may manifest with particular intensity and imbalance in substance use disorder.

The heightened delayed loss avoidance observed in our substance use disorder sample may further reflect altered risk sensitivity. Previous research indicates that substance use disorder individuals often demonstrate elevated risk aversion when confronting potential losses [2, 13], potentially indicating heightened sensitivity and reduced tolerance to negative outcomes. This risk-averse tendency may influence attitudes toward delayed losses in intertemporal choice, motivating preference for delayed negative outcomes to avoid immediate aversive experiences.

In sum, our behavioral findings not only confirm established patterns of immediate reward preference in substance use disorder but crucially demonstrate the context-dependency of decision biases across gain and loss domains. This expansion of our understanding of substance use disorder-related decision-making provides empirical support for theoretical models emphasizing multiple cognitive, emotional, and motivational factors in substance use disorder. The observed loss-avoidance tendencies represent a particularly promising direction for future research employing refined experimental paradigms to further elucidate decision mechanisms across diverse loss scenarios.

### Computational mechanisms underlying context-dependent biases

Our application of the Drift Diffusion Model (DDM) provides mechanistic insights into the cognitive processes underlying the observed behavioral patterns. The drift rate analyses support Hypothesis 2a, revealing distinct information integration patterns between substance use disorder and control participants across contexts. This finding both confirms previous research and extends our theoretical understanding of substance use disorder-related decision processes. Prior studies have demonstrated that substance use disorder-related stimuli capture attentional resources and accelerate information processing [19, 30], consistent with our observations in gain contexts. Importantly, our research reveals that substance use disorder individuals exhibit reversed information integration patterns in loss contexts, with pronounced loss-avoidance tendencies and altered processing rates. This context-dependent processing pattern significantly advances our understanding of cognitive mechanisms in substance use disorder.

The observed context-dependency likely reflects altered motivational states and goal orientations in substance use disorder. Research has established that substance use disorder involves dysregulated reward processing characterized by hypersensitivity to substance-related rewards coupled with diminished responsivity to natural rewards [24]. This reward system imbalance may compromise impulse control in gain contexts, biasing information processing toward immediate reward acquisition. Conversely, in loss contexts, motivational orientation may shift from reward pursuit to loss avoidance, particularly regarding negative consequences associated with substance use (health deterioration, social impairment). This avoidance motivation may direct information processing resources toward delayed loss options, producing seemingly rational decisions. This interpretation aligns with Loewenstein’s (1996) “visceral influences” framework, which emphasizes how goal orientation and motivational states shape information processing strategies and decision preferences.

Our findings align with extensive neuroimaging research documenting prefrontal dysfunction in substance use disorder. Studies by Goldstein and Volkow [24] using fMRI during delay discounting tasks found reduced activation in the dorsolateral prefrontal cortex (dlPFC) in cocaine users, corresponding to the lower decision thresholds observed in our DDM analysis. Similarly, research by Monterosso et al. [54]demonstrated that individuals with methamphetamine dependence showed decreased prefrontal-striatal connectivity during intertemporal choice, consistent with our observed impaired context sensitivity. The reduced drift rates in gain contexts observed in our SUD group may reflect the hypoactivation of the ventromedial prefrontal cortex documented by Peters and Büchel [61] during reward valuation tasks.

Our findings regarding decision thresholds support Hypothesis 2b, revealing generally lower thresholds in substance use disorder participants—a computational signature of impulsivity. This corresponds with extensive evidence that substance use disorder individuals exhibit hastened responding and reduced deliberation in decision tasks [52, 60], potentially stemming from impaired cognitive control and consequence evaluation [24]. Regarding starting point bias (Hypothesis 2c), both groups demonstrated initial preferences for delayed gains and immediate losses, with behavioral differences emerging from subsequent information processing. Our non-decision time results (Hypothesis 2d) indicate that substance use disorder primarily affects central decision processes rather than perceptual encoding or motor execution, partially aligning with findings that cocaine users exhibit prolonged decision times while preserving perceptual and motor functions [66]. However, other studies have identified perceptual processing deficits in substance use disorder [34], suggesting potential variability across substance types and substance use disorder severity that warrants further investigation.

Collectively, our threshold and non-decision time findings suggest that substance use disorder-related decision impairments primarily manifest in central decision processes rather than peripheral perceptual-motor stages. This aligns with the “I-RISA” (Impaired Response Inhibition and Salience Attribution) model [24], which posits dysregulation across multiple neural circuits in substance use disorder: the salience network (ventral striatum, reward sensitivity), memory circuits (hippocampus, amygdala, emotional and habitual responses), and control networks (prefrontal cortex, impulse regulation). Imbalances across these circuits produce the characteristic reward hypersensitivity, craving intensity, and diminished self-control observed in substance use disorder, ultimately generating impulsive, myopic decision patterns. Conversely, perceptual and motor processes remain relatively preserved, likely because they engage primary sensory and motor cortices that appear less disrupted in substance use disorder [24].

Our findings provide systematic support for most hypotheses. Hypothesis 2a (context-specific drift rate differences) was fully supported, with SUD participants showing lower drift rates in gain and higher rates in loss contexts. Hypothesis 2b (lower decision thresholds in SUD) received strong support across contexts. Hypothesis 2c (starting point bias) was partially supported, showing context effects but no group differences. Hypothesis 2d (no non-decision time differences) was confirmed. Task parameter hypotheses (3a-3c) were fully supported, demonstrating the expected monetary and temporal effects and their interaction.

The influence of task parameters (monetary difference and time difference) on decision processes strongly supports Hypotheses 3a-3c. Larger monetary differences accelerated drift rates, reflecting more rapid convergence toward options offering greater monetary value—a finding consistent with research identifying monetary magnitude as a key determinant of intertemporal choice [27, 39]. This effect likely involves dopaminergic activation, with larger monetary amounts potentially triggering greater dopamine release, thereby promoting immediate reward selection [51].

Conversely, larger time differences reduced drift rates, indicating more cautious processing with extended delays—consistent with established temporal discounting effects [26, 37]. This temporal sensitivity may engage prefrontal regions, particularly the dorsolateral prefrontal cortex, which mediates delayed gratification and self-regulatory processes [51]. With extended delays, prefrontal activation may suppress immediate reward impulses, facilitating more deliberative choice.

Most notably, we observed a significant interaction between monetary and temporal parameters, with maximal drift rates occurring when monetary differences were large and time differences small (high short-term value), and minimal drift rates when monetary differences were small and time differences large (low long-term value). This interaction suggests that these parameters operate interdependently rather than independently. Neurobiologically, this interactive effect may reflect competition between striatal regions primarily encoding reward magnitude and prefrontal regions processing temporal information, with relative activation patterns determining choice outcomes [50]. When relative reward per unit time is high, striatal activation predominates, promoting immediate options; when temporal discounting outweighs monetary value, prefrontal activation prevails, facilitating delayed gratification—patterns that align precisely with our behavioral observations.

### Clinical implications

The computational insights provided by our drift diffusion modeling approach suggest promising directions for clinical applications, though translation to practice requires substantial additional validation. The identified DDM parameters—particularly drift rates and decision thresholds—represent promising candidates for biomarkers of substance use disorder severity and treatment response. While traditional approaches to substance use disorder assessment rely heavily on self-report measures and behavioral observations that often lack sufficient objectivity and stability, computational parameters offer more precise quantification of underlying neurocognitive processes. Previous research has established significant associations between DDM parameters and substance use disorder severity [1], suggesting potential diagnostic and prognostic utility. Our context-specific findings further enhance this potential by revealing differential processing patterns across gain and loss domains, potentially offering more nuanced characterization of individual impairments. Future research should systematically evaluate these parameters’ utility within clinical assessment protocols to support diagnosis, relapse prediction, and treatment planning.

Beyond assessment applications, our findings suggest promising directions for intervention development. Conventional substance use disorder treatments typically employ standardized approaches such as pharmacological replacement therapies or generalized cognitive-behavioral interventions with limited consideration of individual neurocognitive profiles. The computational precision offered by DDM analysis enables identification of specific decision-making deficits at the individual level, potentially supporting more personalized intervention strategies. For instance, individuals demonstrating aberrant drift rates might benefit from targeted cognitive training focused on value sensitivity calibration, potentially reducing hypersensitivity to substance-related cues. Similarly, those exhibiting reduced decision thresholds could benefit from interventions specifically targeting cognitive control enhancement and impulse management. Such neurocognitively-informed, personalized approaches could substantially improve treatment precision and efficacy compared to conventional standardized protocols.

However, translating these computational insights into clinical practice requires substantial additional investigation. Future studies must establish the reliability, validity, and specificity of these parameters as biomarkers across diverse clinical populations and contexts. Larger-scale studies incorporating longitudinal designs are needed to determine whether these computational signatures represent stable traits or fluctuating states, and whether they predict clinically meaningful outcomes such as treatment response and relapse vulnerability. Moreover, rigorous randomized controlled trials comparing standard interventions with computationally-informed, personalized approaches are essential to empirically validate the clinical utility of these insights. Such translational efforts necessitate interdisciplinary collaboration spanning cognitive science, computational modeling, neuroscience, and clinical substance use disorder research.

By characterizing the specific computational mechanisms underlying context-dependent decision biases in substance use disorder, this study provides both theoretical advancement and practical clinical direction. The identified parameters offer potential objective metrics for clinical assessment while simultaneously suggesting targeted intervention approaches. These developments represent exploratory pathways toward more precise, personalized, and effective substance use disorder treatment strategies, pending empirical validation through controlled trials.

### Limitations and future directions

Despite its contributions, several methodological constraints warrant consideration when interpreting our findings. Sample representativeness presents a significant limitation, as our focus on female opioid users with specific demographic characteristics may limit generalizability to other substance use disorders or demographic groups. The cross-sectional design precludes determination of whether the observed computational signatures represent antecedents or consequences of substance use disorder. Demographic disparities between groups, particularly the age discrepancy (control: M = 19.3; SUD: M = 24.8), represent potential confounding factors that may influence temporal discounting preferences [69]. The differential incentive structure (cash vs. goods) may have systematically altered decision motivations, as monetary rewards typically hold greater salience for individuals with substance use disorder [38]. Additionally, several difficult-to-quantify factors—including detailed substance use history, withdrawal duration, and comorbid conditions—may have influenced computational parameters [3, 56], introducing alternative explanations for our findings. The rehabilitation center setting may have introduced response biases, while our 10-second decision time limit may have artificially inflated apparent impulsivity. Finally, our neurobiological interpretations rely on inferential links to previous neuroimaging studies rather than direct neural measurements, limiting mechanistic conclusions [21].

Addressing these limitations requires a comprehensive approach encompassing both specific methodological refinements and broader research strategies. Future studies should prioritize several critical improvements: standardizing incentive structures across groups or directly testing motivational differences between cash and goods rewards through controlled experimental comparisons to isolate substance use disorder-specific decision patterns from motivational confounds; and implementing systematic replication across diverse populations—including different substance types, cultural backgrounds, age ranges, and comorbidity profiles—to establish the generalizability of these computational signatures and identify both universal and population-specific mechanisms.

At a broader level, expanded sampling approaches incorporating multi-site data collection across diverse substance use disorders and demographic characteristics would enhance generalizability and identify both universal and substance-specific computational patterns [72]. Methodologically rigorous longitudinal designs with comprehensive assessment of potential moderating variables would help disentangle causal relationships and identify developmental trajectories of computational alterations, tracking individuals from early substance use through various stages of disorder development [59, 70]. From a clinical perspective, prospective studies examining relationships between computational parameters and meaningful outcomes—including treatment response, relapse vulnerability, and functional recovery—are essential to establish predictive validity, while randomized controlled trials comparing standard interventions with computationally-informed approaches are necessary to empirically validate the translational potential of these findings [80]. Through these complementary research approaches, computational methods like the drift diffusion model may transition from theoretical tools to valuable clinical applications in substance use disorder medicine.

## Conclusion

This study employed the Drift Diffusion Model to investigate intertemporal decision-making in female substance use disorder, revealing distinctive context-dependent patterns across behavioral and computational levels of analysis. Behaviorally, the substance use disorder group exhibited stronger preferences for immediate rewards in gain scenarios while demonstrating enhanced avoidance of delayed losses in loss contexts—a pattern further supported by significantly lower discount rates in loss scenarios compared to controls. Computational modeling provided mechanistic insights into these behavioral tendencies, showing that substance use disorder participants maintained consistently lower decision thresholds, indicating impulsive decision characteristics irrespective of context. Importantly, they exhibited context-specific alterations in evidence accumulation processes: lower drift rates in gain scenarios (suggesting reduced sensitivity to non-substance rewards) but higher drift rates in loss scenarios (reflecting heightened sensitivity to negative outcomes). These findings significantly expand our understanding of substance use disorder-related decision mechanisms by highlighting the critical role of contextual factors in shaping choice behavior. By utilizing computational psychopathology methods, this research not only validates and extends existing substance use disorder theories but also identifies potential computational targets for assessment and intervention development, ultimately supporting more personalized approaches to substance use disorder treatment that account for context-dependent decision processes.

These computational insights suggest several therapeutic directions. Cognitive training interventions targeting decision threshold calibration, such as response inhibition training, may help normalize impulsive decision patterns. The context-specific drift rate alterations suggest that treatments should address different mechanisms in gain versus loss contexts—potentially combining reward sensitivity training for gain scenarios with emotion regulation strategies for loss scenarios. For assessment purposes, DDM parameters could serve as objective biomarkers complementing traditional self-report measures, enabling more precise treatment matching and progress monitoring. Future clinical trials should evaluate whether DDM-informed interventions produce superior outcomes compared to standard approaches. It is important to emphasize that while these computational insights suggest promising therapeutic directions, all clinical implications remain exploratory and preliminary. Rigorous validation through controlled trials and longitudinal studies will be essential before these findings can inform clinical practice.

## Data Availability

The datasets generated or analyzed during the current study are publicly available from the corresponding author on request.
